# Non-attendance in mammography screening and women’s social network: a cohort study on the influence of family composition, social support, attitudes and cancer in close relations

**DOI:** 10.1186/s12957-015-0623-5

**Published:** 2015-06-28

**Authors:** Åsa Ritenius Manjer, Ulla Melin Emilsson, Sophia Zackrisson

**Affiliations:** School of Social Work, Lund University, Bredgatan 13, Lund, SE-221 00 Sweden; Department of Translational Medicine, Diagnostic Radiology, Lund University, Lund, Sweden

**Keywords:** Attendance, Breast cancer, Family composition, Health attitudes, Mammography screening, Non-attendance, Social network

## Abstract

**Background:**

Mammography screening can reduce breast cancer mortality. The aim of the present study was to investigate non-attendance in mammography screening in relation to different aspects of a women’s social network, attitudes and cancer in close relations.

**Methods:**

Data from the Malmö Diet and Cancer Study baseline examination in 1991–1996 was used. A re-examination began in 2007, and 1452 women participated. Family composition, social support, sense of belonging, attitudes on screening and breast cancer risk and on previous cancer in close relations were investigated in relation to self-reported participation in mammography screening using logistic regression analysis, yielding odds ratios with 95 % confidence intervals.

**Results:**

Both attendees (98.0 %) and non-attendees (95.2 %) considered mammography screening important. Non-attendance in mammography screening was associated with being unmarried vs. married (2.40:1.30–4.45) and with not having vs. having children (1.77:1.08–2.92). Non-attendees planned to abstain from mammography screening in the future more often than attendees (4.78:2.56–8.90), and they had often abstained from cervical cancer screening (1.69:1.04–2.75). No other statistically significant association was found.

**Conclusions:**

This study indicates that family composition, but not necessarily the presence or absence of social support, perceived cancer risk or cancer in close relations, may affect non-attendance in mammography screening. A positive attitude towards mammography screening was found among both attendees and non-attendees, although the latter group planned to a lesser degree to attend mammography screening in the future.

## Background

Mammography screening, an X-ray examination of the breasts, is used to diagnose breast cancer at an early stage in order to achieve lower breast cancer mortality. Sufficiently high attendance in mammography screening is a key factor to obtain the intended effect. According to European Guidelines, an attendance rate of at least 70 % is required [1]. The Swedish population-based screening programmes have in most parts of the country an attendance rate between 80 and 90 % [2], but in some parts of the country, only 65 % attend, i.e. a 35 % non-attendance rate [3].

It is well established that socio-demographic factors are associated with the risk of non-attendance, but previous studies have mainly reported on traditional factors such as low educational level and manual occupation, and they are associated with a high rate of non-attendance [4–6]. Theories about social network have been extensively studied using different definitions in both medical and social care research [7, 8], and the importance of support from family and friends on a women’s decision to participate in mammography screening has, indeed, been indicated in some previous studies [8–10]. Attitudes about mammography screening in the women’s families and close environment may also affect attendance, but only single studies have been performed [11]. Another factor in close relations (i.e. family and friends), that could influence the decision to attend, is previous cancer or, even, previous breast cancer, but this aspect has not been widely investigated.

Malmö, the third largest city in Sweden, was the base for a randomised trial on mammography screening in the 1970s [12]. A population-based service screening started in 1991. Following this, a large population-based study, The Malmö Diet and Cancer Study, recruited about 17,000 women between 1991 and 1996. All participants answered a questionnaire on lifestyle, socio-economic factors and health behaviour. The cohort has been re-examined at several occasions, and self-reported information on mammography screening is available.

The aim of the present study was to investigate non-attendance in mammography screening in relation to different aspects of a women’s social network: family composition, social support, attitudes and cancer in close relations.

## Methods

### Study population and setting

#### The Malmö Diet and Cancer Study

The Malmö Diet and Cancer Study (MDCS) is a population-based prospective cohort study that recruited women living in Malmö, Sweden, between 1991 and 1996. In all, 17,035 of those invited participated, which corresponded to a participation rate of 40 % [13]. A randomised sample of 3531 women was already at baseline included in a sub-cohort with additional examinations related to cardiovascular risk factors. Out of these 3531 women, the 3045 women who were alive in 2007 were invited to a re-examination. When the re-examination was completed in 2012, 2212 (73 %) of all invited women had participated. The present study included 1554 women who had been re-examined up until 29 September 2010 and who had completed all parts of the baseline examination and the re-examination.

At the baseline examination, 1554 women were between 45 and 68 years of age and 61–84 years at the re-examination. The baseline questionnaire included questions on socio-demographic factors and several questions on the social network [14]. In the re-examination, participants were additionally asked about several aspects of mammography screening. The current study was approved by the regional ethics committee in Lund (Dnr 349/2006 and Dnr 2010/278).

### Mammography screening in the study setting

Mammography screening was introduced in Malmö in 1976 as part of a randomised trial for women between 45–69 years of age [12]. It was followed by the introduction of a population-based mammography screening programme in 1990 where all women between 50–69 years of age were invited every 18 or 24 months depending on age and parenchyma pattern [3]. From 1997, the upper age limit was changed to 74 years, and from 2009 and onwards, the lower limit was changed to 40 years meaning that currently, women aged 40–74 years are invited, in line with the recommendations from the Swedish National Board of Health and Welfare [15]. All women in the eligible age groups are invited by letter, every 18 months for women aged 40–55 years and every 24 months for women >55 years. The fee paid by the attendee for a screening mammogram is 120 SEK (about 12 GBP) and is not reimbursed. For women living in Malmö, the population-based screening has been carried out at Malmö University Hospital, now Skåne University Hospital Malmö.

### Measures and definitions

#### Outcome measure

The outcome variable of interest in this study is mammography screening non-attendance. Based on questions at the re-examination about invitation to mammography screening at Malmö University Hospital and participation in screening at this hospital, the following groups were defined: “invited/ participated”, “invited/did not participate”, “not invited/did not participate” and “not invited/participated”.

“Invited/participated” and “not invited/participated” were classified as “attendees” as all women in the present age groups from the area ought to have received an invitation. Correspondingly, “invited/not participated” and “not invited/not participated” were regarded as “non-attendees”. There were a total of 1452 women who contributed with information on these questions.

### Explanatory factors

#### Baseline questionnaire

The baseline questionnaire included information on country of birth. Educational level was classified into three different levels. Occupation was based on the question “Present or latest job” which had been re-coded into the Swedish socioeconomic classification (SEI) into “Employees, officials/salaried”, “Employees, labourers” and “Employers, self-employed” [14]. Information was also available on marital status, whether the women were living alone or not, and the number of children [14].

The social support factors “Social participation”, “Social anchorage” and “Instrumental support” have previously been defined by Lindström et al. [16]. “Social participation” was based on 13 different yes/no questions about attendance to formal and informal groups in the society during the past year, e.g. a union meeting, going to the cinema or a church meeting. If three alternatives or fewer were indicated, the social participation of that person was considered “low” [16].

“Social anchorage” describes to what extent the person belongs to, and is anchored within, formal and informal groups and the feeling of membership to these groups, familiarity with neighbourhoods, sense of belonging to friends and relatives, membership or position of trust in organisations or clubs and the feelings of being important to other people. It is based on five questions with four possible answers “Yes I am sure”, “Yes probably”, “No probably not” and “No not at all” that were dichotomised to yes/no answers. The two highest options were set against the two lowest. If three or more of the five items denoted low social anchorage, the whole variable was regarded as “low” [16].

“Instrumental support” was based on one question with four alternative answers on their ability to get help from people if they fall ill or need help with practical things. The women who responded that there is certainly help available were classified as “High” and the other three alternatives were classified as “low” [16].

### Re-examination questionnaire

“Planned future participation in mammography screening” had three alternative answers: “yes”, “no” and “don’t know”. “Self-rated risk for getting breast cancer” was rated as “low”, “medium” and “high”. “Attitude on mammography screening” was assessed by the question: “what do you think about mammography screening” and had three alternative answers: “it is good—it improves the chance to recover from breast cancer”, “it makes no difference—does not affect my health” and “it does more harm than good—can be dangerous”. “Previous cervical screening” was defined from the question: “have you made a gynaecological health control with pap smear test” with alternative answers “yes” and “no”.

“Cancer in close relationships” was based on four different yes/no questions about cancer: “mother has/has had cancer”, “father has/has had cancer”, “siblings have/have had cancer” and “close relatives, friends, fellow workers have/have had cancer”. If at least one person in the surrounding has or has had cancer, this was classified as a “cancer in close relations”. Accordingly, “breast cancer experience” was based on three questions about breast cancer: “mother has/has had breast cancer”, “sister has/has had breast cancer” and “close relatives, friends, follow workers have/had have breast cancer”. If at least one person in the surrounding has or has had breast cancer, this was classified as a “breast cancer in close relations”. “Previous cancer” and “previous breast cancer” in the respondents’ medical history were assessed by yes/no questions.

### Statistical methods

The risk of “non-attendance” in relation to various factors was calculated using logistic regression analysis. Odds ratios (OR), for “non-attendance” with 95 % confidence intervals (CI) were calculated. All variables were adjusted for age at the time of re-examination in a second model. Many studies have found non-attendance to be associated with age, country of birth, educational level and occupation. Subsequently, a final model included these co-variates. Several of the studied factors can be expected to be strongly correlated, e.g. “marital status” and “living alone”, and it was not considered suitable to perform an analysis including all studied factors in the same model. SPSS 17.0 was used for all analyses.

## Results

### Socio-demographic factors

These factors were selected a priori to be included in the final statistical model. There were no large differences in age (mean: 74.1 vs. 72.7 years) or foreign background (12.9 vs. 9.2 %) between non-attendees and attendees. Non-attendees had worked to a greater extent as labourers (43.5 vs. 35.5 %), and had a shorter education (a maximum of 10 years: 76.6 vs. 70.5 %), as compared to attendees (Table 1).

**Table 1:** Socio-demographic factors in attendees and non-attendees

Factors	Category	Attendees number (%)	Non-attendees number (%)
Age (years)		*72.7* (*5.5*)	*74.1* (*6.0*)
Born in Sweden	Yes	1205 (90.7)	108 (87.1)
	No	122 (9.2)	16 (12.9)
	Missing	1 (0.1)	0
Level of education	University	295 (22.2)	19 (15.3)
	11–12 years	97 (7.3)	10 (8.1)
	10 years	936 (70.5)	95 (76.6)
	Missing	0	0
Occupation	Officials/salaried	762 (57.4)	62 (50.0)
	Labourers	472 (35.5)	54 (43.5)
	Self-employed	87 (6.6)	7 (5.56)
	Missing	7 (0.5)	1 (0.8)

Mean and SD are in italics

### Family composition and social support

Women who were unmarried vs. married (adjusted OR = 2.40:1.30–4.45), and who had no children vs. children (1.77:1.08–2.92), were at a statistically significant higher risk for not attending mammography screening (Table 2). Non-attendees were more often divorced or widowed (1.31:0.87–1.97) and lived alone more often (1.30:0.89–1.90) as compared to attendees, but these associations were not statistically significant (Table 2). Social participation was not associated with non-attendance. A low social anchorage (1.32:0.71–2.43) and a low instrumental support (1.21:0.81–1.83) were positively associated with non-attendance, but these estimates did not reach statistical significance (Table 2).

**Table 2:** Social network and family composition: the risk of not attending mammography screening

Factor	Category	Attendees	Non-attendees	OR (95 % CI)	OR (95 % CI)	OR (95 % CI)
		*N* (%)	*N* (%)	Crude	Age-adjusted	Adjusted^a^
Marital status	Married	635 (47.8)	46 (37.1)	1.00 (reference)	1.00 (reference)	1.00 (reference)
	Unmarried	104 (7.8)	16 (12.9)	2.12 (1.16–3.90)	2.26 (1.23–4.16)	2.40 (1.30–4.45)
	Divorced/widow	581 (44.8)	61 (49.2)	1.45 (0.97–2.16)	1.34 (0.90–2.01)	1.31 (0.87–1.97)
	Missing	8 (0.6)	1 (0.8)	–	–	–
Children	Yes	1132 (85.2)	99 (79.8)	1.00 (reference)	1.00 (reference)	1.00 (reference)
	No	150 (11.3)	22 (17.7)	1.69 (1.03–2.74)	1.74 (1.06–2.85)	1.77 (1.08–2.92)
	Missing	46 (3.5)	3 (2.4)	–	–	–
Living alone	No	710 (53.5)	56 (45.2)	1.00 (reference)	1.00 (reference)	1.00 (reference)
	Yes	609 (45.9)	68 (54.8)	1.42 (0.98–2.05)	1.31 (0.90–1.91)	1.30 (0.89–1.90)
	Missing	9 (0.7)	0	–	–	–
Social participation	High	983 (74.0)	88 (71.0)	1.00 (reference)	1.00 (reference)	1.00 (reference)
	Low	343 (25.8)	36 (29.0)	1.17 (0.78–1.76)	1.05 (0.71–1.63)	0.95 (0.62–1.47)
	Missing	2 (0.2)	0	–	–	–
Social anchorage	High	1218 (91.7)	111 (89.5)	1.00 (reference)	1.00 (reference)	1.00 (reference)
	Low	110 (8.3)	13 (10.5)	1.30 (0.71–2.38)	1.36 (0.74–2.51)	1.32 (0.71–2.43)
	Missing	0	0	–	–	–
Instrumental support	High	997 (75.1)	87 (70.2)	1.00 (reference)	1.00 (reference)	1.00 (reference)
	Low	328 (24.7)	37 (29.8)	1.29 (0.86–1.94)	1.25 (0.83–1.87)	1.21 (0.81–1.83)
	Missing	3 (0.2)	0	–	–	–

^a^Socioeconomic factors: age, born in Sweden, level of education and occupation

### Health attitudes and screening behaviour

In all, 95.2 % of non-attendees and 98.0 % of attendees considered mammography screening to be important and that it improves the chance of recovery from breast cancer (Table 3). That is, very few women considered that screening “makes no difference”. However, the small difference resulted in an about four times higher OR concerning non-attendance in these women, but with a very wide CI (adjusted OR = 4.04:1.38–11.83). The self-rated risk of breast cancer was not associated with attendance. The intention not to participate in mammography screening in the future was strongly associated with the risk of non-attendance (4.78:2.56–8.90), and women who had previously abstained from cervical screening were at a statistically significant high risk of being non-attendees in mammography screening (1.69:1.04–2.75) (Table 3).

**Table 3:** Health behaviour and the risk of not attending mammography screening

Factor	Category	Attendees	Non-attendees	OR (95 % CI)	OR (95 % CI)	OR (95 % CI)
		*N* (%)	*N* (%)	Crude	Age-adjusted	Adjusted^a^
Attitude on mammography screening	It is good	1302 (98.0)	118 (95.2)	1.00 (reference)	1.00 (reference)	1.00 (reference)
	It makes no difference	12 (0.9)	5 (4.0)	4.60 (1.59–13.27)	4.21 (1.45–12.22)	4.04 (1.38–11.83)
	It does more harm than good	2 (0.2)	0	–	–	–
	Missing	12 (0.9)	1 (0.8)	–	–	–
Self-rated risk of breast cancer	Low	750 (56.5)	68 (54.8)	1.00 (reference)	1.00 (reference)	1.00 (reference)
	Medium	488 (36.7)	49 (39.5)	1.11 (0.75–1.63)	1.18 (0.80–1.75)	1.20 (0.81–1.79)
	High	56 (4.2)	5 (4.0)	0.98 (0.38–2.54)	1.10 (0.42–2.86)	1.10 (0.42–2.88)
	Missing	34 (2.6)	2 (1.6)	–	–	–
Planned future participation	Yes	1232 (92.8)	87 (70.2)	1.00 (reference)	1.00 (reference)	1.00 (reference)
	No	46 (3.5)	16 (12.9)	4.93 (2.68–9.75)	4.74 (2.57–8.74)	4.78 (2.56–8.90)
	Do not know	43 (3.3)	12 (9.7)	3.95 (2.01–7.77)	3.47 (1.74–6.89)	3.37 (1.69–6.72)
	Missing	7 (0.5)	9 (7.3)	–	–	–
Previous cervical screening	Yes	1143 (86.1)	96 (77.4)	1.00 (reference)	1.00 (reference)	1.00 (reference)
	No	156 (11.7)	26 (21.0)	1.98 (1.25–3.16)	1.71 (1.06–2.78)	1.69 (1.04–2.75)
	Missing	29 (2.2)	2 (1.6)	–	–	–

^a^Socioeconomic factors: age, born in Sweden, level of education and occupation

### Cancer in family and close relations

The experience of cancer or breast cancer in the own family or in close relations was not associated with the risk of non-attendance (Table 4). Women who had themselves experienced a cancer diagnosis had a similar risk of non-attendance as women with no cancer history, corresponding to an OR of 1.16 (0.77–1.75). Women who had a previous history of breast cancer had a decreased risk of non-attendance, but this association was not statistically significant (0.41:0.15–1.15).

**Table 4:** Cancer in close relations and the risk of not attending in mammography screening

Factor	Category	Attendees	Non-attendees	OR (95 % CI)	OR (95 % CI)	OR (95 % CI)
		*N* (%)	*N* (%)	Crude	Age-adjusted	Adjusted^a^
Cancer in family	No	647 (48.7)	57 (46.0)	1.00 (reference)	1.00 (reference)	1.00 (reference)
	Yes	681 (51.3)	67 (54.0)	1.12 (0.77–1.61)	1.12 (0.77–1.62)	1.10 (0.76–1.60)
	Missing	0	0	–	–	–
Cancer in close relations	No	656 (49.4)	62 (50.0)	1.00 (reference)	1.00 (reference)	1.00 (reference)
	Yes	672 (50.6)	62 (50.0)	0.98 (0.68–1.41)	1.04 (0.72–1.51)	1.07 (0.74–1.56)
	Missing	0	0	–	–	–
Breast cancer in family	No	1144 (86.1)	104 (83.9)	1.00 (reference)	1.00 (reference)	1.00 (reference)
	Yes	184 (13.9)	20 (16.1)	1.20 (0.72–1.98)	1.17 (0.71–1.94)	1.18 (0.72–1.97)
	Missing	0	0	–	–	–
Breast cancer in close relations	No	761 (57.3)	74 (59.7)	1.00 (reference)	1.00 (reference)	1.00 (reference)
	Yes	567 (43.1)	50 (40.3)	0.91 (0.62–1.32)	0.98 (0.67–1.43)	1.04 (0.71–1.53)
	Missing	0	0	–	–	–
Have had cancer	No	994 (74.8)	89 (71.8)	1.00 (reference)	1.00 (reference)	1.00 (reference)
	Yes	334 (25.2)	35(28.2)	1.17 (0.78–1.76)	1.16 (0.77–1.75)	1.16 (0.77–1.75)
	Missing	0	0	–	–	–
Have had breast cancer	No	1224 (92.2)	120 (96.8)	1.00 (reference)	1.00 (reference)	1.00 (reference)
	Yes	104 (7.8)	4 (3.2)	0.39 (0.14–1.08)	0.40 (0.14–1.10)	0.41 (0.15–1.15)
	Missing	0	0	–	–	–

^a^Socioeconomic factors: age, born in Sweden, level of education and occupation

## Discussion

This study demonstrated that family composition may affect non-attendance in mammography screening. Virtually, all women, attendees and non-attendees, considered mammography screening to be important. Non-attendees planned to a lesser degree to attend mammography screening in the future, and had relatively often abstained from cervical screening in the past, which indicates that their decisions were not a random phenomenon.

### Family composition and social support

#### Non-attendance was more common in women living alone and in women with no children

Decision-making in a social context was illustrated by Willis showing that women felt a responsibility to attend when they got an invitation [17]. It is possible to hypothesise that this effect may be more pronounced in women living together with someone else or having children.

In the present study, there were no differences between women who did not attend and those who attend mammography screening with regard to active social participation. It might indicate that the decision not to attend cannot directly be linked to how active women are in the society in general. On the other hand, it may be a result of the MDCS population being a subset of the general population that is more active, as indicated by the fact that they decided to take part of the MDCS, while the result might have been different in the general population.

The present study did not show statistically significant results regarding instrumental support and social anchorage. There was a tendency towards differences between the groups where the experience of affinity with other people (“high social anchorage”), and the ability to get help from someone in the social network (“high instrumental support”), may have an impact on attendance in mammography screening. The non-significant results can be an effect of the small sample size. On the other hand, there is no data available regarding the extent and the impact of close relations outside the family. However, previous studies have shown that informal interpersonal aspects, like trust to someone in the network, can be important and can create good conditions for health [7, 8, 17].

### Health attitudes and screening behaviour

Non-attendees in mammography screening planned to abstain from mammography screening in the future, and had abstained from cervical screening, to a greater extent than women who had attended mammography. Similar results have also been seen in previous research which shows a clear influence from past screening behaviour on future attendance in mammography screening [11]. The fact that almost all women in this study answered that mammography screening improves their possibility of getting cured of breast cancer, even those who did not attend mammography screening, also suggests that this is a planned action.

Women’s intention to attend future mammography screening may also be based on a moral desire to live a healthy life and be a responsible citizen who has done everything possible to detect breast cancer in early stage [17]. Research on attendees in cervical screening in Sweden shows results in the same direction [18].

### Cancer in family and close relations

There were no differences between attendees and non-attendees in mammography screening regarding the experience of cancer or breast cancer in the family and the close relationships.

This is in contrast to previous studies which shows that a history of cancer in the family or among close friends were important for the decision to attend mammography screening [8, 17].

The result in the present study can indicate trust to the health care system and experiences of good examples where women have been cured from cancer, but this issue needs further attention, preferably in qualitative studies.

### Strengths and limitations of the study

This is a prospective follow-up of a large population-based cohort, and national population registries make it easy to follow the population over time. The study was performed in a population that has been exposed to a general service screening during the last 20 years. An additional strength is that the questionnaire contains information that makes it possible to adjust the analyses for potential confounders.

Some aspects of study limitations should be considered. In the MDCS, the educational level of the participants is slightly higher than that in the general population and the percentage of foreign-born women is lower in this material than that in Malmö in general, which could limit the representativeness [13]. However, as there was a wide distribution in the studied socio-demographic factors, internal comparisons, e.g. relative risks, were probably not affected to any large extent by a potential selection bias. A problem in some of the analyses is the low number of individuals, hence a low statistical power. This may have lead to a type II error in relation to some of the analysed factors.

Another limitation is that some of the comparisons are cross-sectional, that is, factors only assessed at the re-examination were indeed measured following the decision concerning participation in mammography screening. Therefore, it is difficult to investigate a potential cause-effect relation, and these comparisons ought to be regarded as descriptive and explorative.

## Conclusions

This study indicates that family composition, but not necessarily the presence or absence of social support, perceived cancer risk or cancer in close relations, may affect non-attendance in mammography screening. A positive attitude towards mammography screening was found among both attendees and non-attendees, although the latter group planned to a lesser degree to attend mammography screening in the future, and had relatively often abstained from cervical screening in the past. The decision not to attend seems not to be a random phenomenon, although our results indicate a complex pattern behind non-attendance, which needs to be further elucidated.
